# Heterogeneous Distribution of Fetal Microchimerism in Local Breast Cancer Environment

**DOI:** 10.1371/journal.pone.0147675

**Published:** 2016-01-25

**Authors:** Dragos Nemescu, Ramona Gabriela Ursu, Elena Roxana Nemescu, Lucian Negura

**Affiliations:** 1 Department of Obstetrics and Gynecology, University of Medicine and Pharmacy "Gr.T.Popa", Iasi, Romania; 2 “Cuza Voda” Obstetrics and Gynecology Hospital, Iasi, Romania; 3 Department of Microbiology, University of Medicine and Pharmacy "Gr.T.Popa", Iasi, Romania; 4 Department of Infectious Diseases, University of Medicine and Pharmacy "Gr.T.Popa", Iasi, Romania; 5 Department of Immunology and Oncogenetics, University of Medicine and Pharmacy "Gr.T.Popa", Iasi, Romania; Meharry Medical College, UNITED STATES

## Abstract

Fetal cells enter maternal circulation during pregnancy and persist in the woman’s body for decades, achieving a form of physiological microchimerism. These cells were also evidenced in tumors. We investigated the frequency and concentration of fetal microchimerism in the local breast cancer environment. From 19 patients with confirmed breast neoplasia, after breast surgical resection, we collected three fresh specimens from the tumor core, breast tissue at tumor periphery, and adjacent normal breast tissue. The presence of male DNA was analyzed with a quantitative PCR assay for the sex determining region gene (SRY) gene. In the group of women who had given birth to at least one son, we detected fetal microchimerism in 100% of samples from tumors and their periphery and in 64% (9 of 14) of those from normal breast tissue. The tissues from the tumor and its periphery carry a significantly increased number of SRY copies compared to its neighboring common breast tissue (p = 0.005). The median of the normalized SRY-signal was about 77 (range, 3.2–21467) and 14-fold (range, 1.3–2690) greater in the tumor and respectively in the periphery than in the normal breast tissue. In addition, the relative expression of the SRY gene had a median 5.5 times larger in the tumor than in its periphery (range, 1.1–389.4). We found a heterogeneous distribution of fetal microchimerism in breast cancer environment. In women with sons, breast neoplasia harbors male cells at significantly higher levels than in peripheral and normal breast tissue.

## Introduction

Studies uniformly have shown that fetal cells are transferred into the maternal circulation during pregnancy, in humans and other mammalian species [1]. Although most of them disappear after birth, some populations persist in the maternal tissues and circulation, without any apparent graft-versus-host reaction or graft rejection. This phenomenon, called fetal cell microchimerism, is present in women for decades after pregnancy [2]. It involves various cell types including multi-lineage stem cells [1]. Fetal cells migrated into the affected maternal tissues, differentiate into phenotypes as renal, hepatic or nervous tissue, mainly to support the tissue repair process [1]. Moreover, in the inflammatory lesions which occur during pregnancy, these fetal cells, as endothelial precursors, are able to form blood vessels that express CD31 and/or VEGFR2 [3].

Microchimeric fetal cells were observed in almost all normal tissues of women with sons [4, 5]. These cells remain functional and are involved in pathological processes, in autoimmune and degenerative diseases [6]. They were also detected into the maternal stroma of various human neoplastic tissues: thyroid, lung, melanoma, cervix and breast [7–10].

Breast cancer microchimerism was evaluated in peripheral blood and breast tissue. PCR amplification of fetal SRY gene from maternal blood found an increase in the number of chimeric cells in healthy patients compared to those with breast cancer [11–13]. The authors conclude that fetal microchimerism has a role of protection against breast cancer through an immune mechanism in the blood. This offers a potential explanation for the classic epidemiologic studies which shown that the increased number of births reduces the incidence of breast cancer [14].

Few studies evaluated the fetal microchimerism at the level of neoplastic breast tissue in humans. Two case-control studies found that in breast samples from healthy women, the microchimerism prevalence was higher than in those from breast neoplasia [15, 16]. The third study assessed fetal cells in almost all (90%) samples from breast carcinoma and none in benign lesions. These were distributed on the entire area of the specimens, preferentially within the tumoral area [8]. However, these studies either did not consider the women reproductive history (male pregnancies or sons)[15, 16], used breast tissue fixed in formaldehyde and embedded in paraffin [8, 15], or were performed on neoplastic tissues associated to pregnancy [8].

Therefore, the aim of our study was to specify the frequency and concentration of fetal microchimerism in the neoplastic breast tissue and its environment, on probes not fixed, not related with pregnancy, considering also the reproductive history of patients.

## Materials and Methods

### Patients and samples

Women scheduled for breast cancer surgery were prospectively recruited to contribute with excess tissue from surgery into this study. We included women with confirmed breast malignancy by pathology and no preoperative chemotherapy, radiotherapy or endocrine therapy specifically given as treatment for breast cancer. At the time of initial diagnosis, written informed consents were obtained from all the patients, and their relevant clinically data were recorded on a pre-defined questionnaire. The study protocol was approved by the Institutional Ethics Committee (“Cuza Voda” Obstetrics & Gynecology Hospital).

After mastectomy or lumpectomy, the pathologist collected three fresh samples from the tumor core, breast tissue at tumor periphery and adjacent normal, not involved breast tissue. Each sample was taken using a disposable biopsy punch with plunger system (3 mm diameter, Kai Industries, Seki, Japan) and stored at 4°C in cryovials with RNAlater stabilization agent (Sigma-Aldrich, St. Louis, MO, USA) until laboratory transfer, where was kept at -80°C. In order to prevent male DNA contamination, pathological specimen manipulation and sampling was performed by female technicians only, blinded to the clinical data of the patients. We followed standard protocols to prevent contamination (single use biopsy punches) and we also changed needles, blades and tips between sample dissections.

### Genomic DNA extraction

Frozen breast tissue samples about 50 mg were thawed. We cut tissues of about 25 mg in little pieces with sterile scalpel blade, suspended in 80 μl PBS and immediately disrupted using TissueRuptor (Qiagen, Valencia, CA; USA) system to decrease lysis time. Tissue homogenates were incubated in a shaking water bath for 3 hours at 56°C with 12 mAU proteinase K (Qiagen, Valencia, CA; USA). Total DNA was extracted using the QIAamp DNA Mini Kit (Qiagen, Valencia, CA; USA) according to the manufacturer’s protocol. DNA was eluted in a 50 μl final volume and stored at 4°C for a maximum of 24 hours before PCR amplification. The extracted DNA was quantified comparatively using a 20X dilution on a Biowave DNA Lifescience Spectrophotometer (Biochrome, Cambridge, UK), or without dilution on the NanoPhotometer Pearl (Implen, Munchen, Germany). The quantification results were similar. We obtained 30–65 ng/μl DNA per probe, with an A260/A280 ratio of 1.8–1.9.

### Quantitative Y-chromosome detection

We detected genetic material transferred from the fetus to the mother using as a fetal marker the sex determining region gene (SRY) from on the Y-chromosome [17]. The levels of the human male SRY (Chromosome: Y; Location: Yp11.3; GeneID: 6736) were assessed using a quantitative real-time PCR method with a Quantifiler^®^ Duo DNA quantification kit (Life Technologies, Carlsbad, CA, USA) on an Applied Biosystems 7500 Fast DX Real-Time PCR system (Life Technologies, Carlsbad, CA, USA) according to the manufacturer’s protocol [18]. The results were analyzed by Applied Biosystems 7500 Fast DX Real-Time software (Life Technologies, Carlsbad, CA, USA) and the human autosomal target ribonuclease P RNA component H1 was used as an endogenous reference for normalizing the measured levels of SRY (RPPH1; Chromosome: 14; Location: 14q11.2; GeneID: 85495)[18]. The relative expression ratio of the SRY gene in tumor samples versus tumor periphery and adjacent normal breast tissue, according to the levels of the RPPH1, was calculated as described previously [19].

The Quantifiler^®^ Duo DNA assay uses two standard quantitation curves: human DNA (target: RPPH1, amplicon 140 bp) and male DNA (target: SRY, amplicon 140 bp). We achieved a quantitative linear correlation from 23 pg/μl to 50 ng/μl of male genomic DNA, in the presence of the same concentration of human DNA (S1 Fig).

The kit can reproducibly detect quantities as low as 5.75 pg/μL for the human assay (limit of detection), with some variation for the SRY specific assay due to its haploid nature [20]. The kit can also accurately quantify 23 pg/μL of human genomic DNA in a sample (limit of quantification). When 2.0 μL of a sample at this concentration is loaded in a reaction, the well contains only nearly 7 diploid human genome equivalents. These equivalents correspond to about 14 copies of the RPPH1 target and approximately seven copies of the SRY target [20].

For samples from the adjacent normal, not involved breast tissue, the Real-Time PCR reactions were performed in triplicate. A specimen was considered positive for microchimerism if it was found at least one copy of male DNA. The amounts of DNA in ng were converted to genome equivalents (gEq)/ml using a factor of 6.6 pg of DNA per cell [21]. Within the experiment, a No Template Control was performed with water instead of DNA. These and negative controls generate no signal for the amplification of male DNA.

### Statistics

All statistical analyses were performed using the SPSS program, version 21.0 (SPSS, Chicago, IL). The differences in SRY expression between the tumor and surrounding tissues were analyzed using the Fisher’s exact test, Wilcoxon signed-rank test for two related samples or paired samples Student’s t-test, as appropriate. For all parameters, a two-sided p <0.05 was considered to be statistically significant.

## Results

Breast cancer tissues and adjacent tissues were obtained from 19 patients who underwent breast surgical resection between January 2013 and June 2014. Theirs clinical characteristics are summarized in Table 1.

**Table 1:** Clinical characteristics of patients with breast cancer.

Characteristic	n = 19
**Mean age at surgery (range)**	62.4 (36–75)
**Reproductive history (women with sons)**	
** Mean number of pregnancies (range)**	4.5 (1–9)
** Mean number of deliveries (range)**	2.1 (1–4)
** Mean number of sons (range)**	1.8 (1–3)
**Postmenopausal**	15 (79%)
**AJCC stage**	
** I**	4 (21%)
** II**	10 (52.6%)
** III**	5 (26.3%)
**Histology**	
** Ductal**	12 (63.2%)
**Mixed, ductal and lobular**	7 (36.8%)
**Bloom Richardson grade**	
**1**	4 (21%)
**2**	11 (58%)
**3**	4 (21%)
**Positive estrogen receptors**	16 (84%)
**Positive progesterone receptors**	15 (79%)
**HER2 positive**	0 (0%)

Data are shown as mean (range) or number (%). A specimen was considered receptor positive if >5% of the cells shows receptors.

According to their obstetrical history, our patients could be stratified in three groups: without any pregnancy (two cases); without any male delivery but abortions (three cases) and with at least one male child (14 cases).

Two of our cases, without any pregnancy in their history, did not show any amplification of the SRY gene, for all samples from any position into the breast and confirm our negative controls.

On three patients, without any male child, but with abortions in their history, we found the presence of microchimerism in two samples from the tumor, one sample from periphery and none in adjacent normal, not involved breast tissue. Due to the low concentrations of male DNA, the quantification was possible only for tumor samples and showed 20, 34 copies of the SRY target. The mean diploid human genome equivalents tested, as determined with RPPH1 quantitative PCR, for all tissue samples in this group, was 9.42 x 10^6^ (range, 6.76 x 10^4^ to 3.05 x 10^7^gEq/ml).

For women who had given birth of at least one son, we detected fetal microchimerism in 100% of samples from tumors and their periphery and in 64% (9 of 14) of those from normal breast tissue. The characteristics of fetal microchimerism at the level of breast cancer and its local environment for this group are presented in Table 2 (number of SRY copies, relative expression ratio and diploid human genome equivalents).

**Table 2:** Characteristics of fetal microchimerism for women who had given birth of at least one son.

	Tumor	Periphery	Normal breast
**SRY-positive**	14 (100%)	14 (100%)	9 (64%)
**SRY copies**	171.5 (32–2454)	86 (8–1113)	7 (0–7)^a^
**RelExpRatio (vs. normal)**	76.7 (3.2–21467)	14.3 (1.3–2690)	1
**RelExpRatio (vs. periphery)**	5.5 (1.1–389.4)	1	-
**Total human, x10**^**5**^**gEq/ml**	79 (5.4–1032)	153.5 (30.2–522)	28.9 (3.9–438)

Data are presented as count (%) or median (range). RelExpRatio: relative expression ratio. gEq: genome equivalents.

^a^All, with except one samples from the normal breast tissue, had the concentration of male DNA below the limit of quantification (23 pg/μl).

There are significant differences in the distribution of the microchimerism between samples from the tumor core, periphery and normal breast tissue, respectively (Fisher’s Exact test, p = 0.04). Moreover, tissues from the tumor and its periphery carry a significantly increased number of SRY copies compared to the neighboring normal breast tissue (Wilcoxon Signed-Rank test for two related samples, n = 14, p = 0.005). The median of the normalized SRY-signal was about 77 and 14-fold greater in the tumor and respectively in the periphery, than in the normal breast tissue. In addition, microchimeric cells are 5 to 6 times more frequent (median) in tumor than in its periphery, but their number in neighboring normal breast tissue is very low, and they are not always identifiable. Total human DNA, determined with ribonuclease P RNA component H1, was significantly higher in tumor periphery compared with normal breast tissue (paired samples t-test, df = 13, p = 0.02).

## Discussion

This is the first study where quantification of breast cancer microchimerism was stratified according to the obstetrical history: the number of sons. Women without any pregnancy did not show microchimeric cells on any of the three samples of the breast. For those with abortions in their history, but without delivery of any male child, low levels of SRY amplification were present in some breast probes, especially from the tumor and its periphery. In this group, normal breast tissue was microchimeric-negative on all patients. We detected SRY target in all samples from the tumor and its periphery, in case of women who delivered at least one male child. In this group, normal breast tissue was positive for microchimerism in 64% of cases.

Although the presence of microchimeric cells was signaled in tumors, there are only three studies in humans to evaluate this phenomenon directly on breast neoplastic tissues, and with discordant results.

One study compared breast tissue minimally involved (<10% involvement) from patients with invasive breast neoplasia and normal breast tissue from mammoplasties [16]. Even if the reproductive history was not available, the authors concluded that common breast tissue from women without cancer frequently contains more microchimeric cells than minimally involved tissue adjacent to an invasive breast cancer (63% vs. 26%). Also, they observed a large variation of the male chimeric cells within the breast cancer group, from 0 to 160 genome equivalents per 10^6^ breast cells.

Dubernard et al.[8] identified microchimeric cells in breast tumors from pregnant women with male fetuses diagnosed with ductal carcinoma during pregnancy or six months postpartum [8]. Using FISH and immunochemistry techniques on pathological samples fixed in formaldehyde and embedded in paraffin, they found that 90% (9 out of 10) samples from ductal carcinoma contained microchimeric fetal cells and none in the four benign lesions. The number of microchimeric cells within the tumor varied from 7 to 165 per 10^6^ breast cells. Furthermore, they noted, without a precise quantification, that fetal cells were distributed on the entire area of the specimens, but were situated preferentially in the tumoral area.

A third study assessed the level of microchimerism in breast samples fixed in formaldehyde and embedded in paraffin, using an in-house quantitative PCR technology oriented towards the testis-specific protein Y-encoded genes [15]. The reproductive history was not available. The authors found that only 21% of neoplastic samples were positive for the Y chromosome compared to 55% of the control cases. Additionally, was identified a cohort of patients with breast neoplasia who had a significantly increased number (>16-fold) of male fetal cells compared to the normal tissue, a phenomenon called by the authors “hyperchimerism”.

Some studies did not consider the reproductive history. Thus, we cannot compare our microchimerism frequency with their results [15, 16]. However, all our samples from the tumor were SRY-positive, comparing with 21% reported by Dimholea et al [15]. This is a striking difference which could appear by using non-fixed breast tissues, only from women with sons, analyzed with a validated method. Moreover, using real-time PCR, we observed the same high frequency of microchimerism in tumor (100%) like Dubernard et al, but at higher concentration [8]. We confirmed samples with a high chimeric presence (X1000 SRY copies) at the level of breast tumors and their periphery [15].

The heterogeneous distribution of the chimeric cells in local breast cancer environment can occur through their recruitment by tumor tissue, local inflammation or an unknown factor. This reduces simultaneously the number of chimeric cells from the woman’s body, as was observed in peripheral blood and breast tissue. Previous studies showed that fetal microchimerism is significantly rare in peripheral blood of women with in situ [13] or invasive breast cancer [11, 12], comparing with normal controls. Microchimeric cells are significantly less frequent in minimally involved tissue next to an invasive breast cancer compared to breast tissue from women without cancer [16]. Our study demonstrated also that there is a very low number of SRY copies in normal breast tissue from the environment of an invasive breast cancer. Thus, the absence of microchimerism seems to be a result of a developing breast cancer. This is supported by recent studies, which found that male microchimerism in peripheral blood is associated with a reduction of breast cancer risk by a third [22] and a 60% lower all-cause mortality, compared with SRY-negative women [23]. The phenomenon appears to be specific to breast cancer, since it is not associated with a mortality reduction in colon cancer [24]. Also, because the microchimerism decrease in peripheral blood appears years before the diagnosis of breast tumor, it could be used in an early detection of breast cancer.

## Conclusions

This study demonstrated that fetal microchimerism seems to be always present in breast tumors from patients with a previous male child. This phenomenon has a heterogeneous distribution at the level of breast cancer environment: tumor core, periphery and normal breast tissue. Thus, the tumor and its periphery harbor SRY-positive cells at higher levels than in normal breast tissue. There is a continuous decrease of the microhimerism concentration from the tumor, to periphery and normal breast tissue, respectively. Future studies should aim to determine the mechanism by which these cells are recruited in the tumor and their influence in neoplastic tissue.

## Supporting Information

S1 Appendix Mathematical model for relative quantification. (PDF)

S1 Fig Male and human quantification standard linearity analysis (example). (PDF)

S2 Fig Amplification plot for human and male DNA, for standards and samples (example). (PDF)
