# Supplementary material for: Heterogeneous Distribution of Fetal Microchimerism in Local Breast Cancer Environment
Source: PLoS One. 2016 Jan 25;11(1):e0147675. doi: 10.1371/journal.pone.0147675 (PMC4726590; doi:10.1371/journal.pone.0147675)
Supplement: S1 Appendix — (PDF) [file pone.0147675.s001.pdf]

S1 Appendix. Mathematical model for relative quantification.

The relative expression ratio of the SRY gene in tumor samples versus tumor periphery and adjacent normal breast tissue, in comparison to the levels of Ribonuclease P RNA Component H1 (RPPH1) reference gene, was calculated through Equation 1 (Pfaffl et al, 2001).

$$\text{Ratio}= \frac{(E_{target})^{\Delta CP_{target}(control\text{-}sample)}}{(E_{ref})^{\Delta CP_{ref}(control\text{-}sample)}} \quad (1)$$

E_{target} is the real-time PCR efficiency of target gene transcript; E_{ref} is the real-time PCR efficiency of a reference gene transcript; ΔCP_{target} is the CP deviation of control – sample of the target gene transcript; ΔCP_{ref} = CP deviation of control – sample of reference gene transcript. Crossing point (CP) is defined as the point at which the fluorescence rises appreciably above the background fluorescence.
