# Supplementary material for: Heterogeneous Distribution of Fetal Microchimerism in Local Breast Cancer Environment
Source: PLoS One. 2016 Jan 25;11(1):e0147675. doi: 10.1371/journal.pone.0147675 (PMC4726590; doi:10.1371/journal.pone.0147675)
Supplement: S1 Fig — (PDF) [file pone.0147675.s002.pdf]

S1 Figure. Male and human quantification standard linearity analysis (example).

Linearity analysis of SRY (target: Duo Male) and RPPH1 (target:Duo Human) PCR standard curve. The graphs were generated by the 7500 SDS v1.2.3 software of Applied Biosystems. The Ct values were collected at the threshold line (S2 Fig). The gradient consisted of 8 dilutions of male and human genomic DNA standard with decreasing template DNA: 50ng, 16.7 ng, 5.56 ng, 1.85 ng, 0.62 ng, 0.21 ng, 68 pg, 23 pg. The non-templated controls generated no signal.

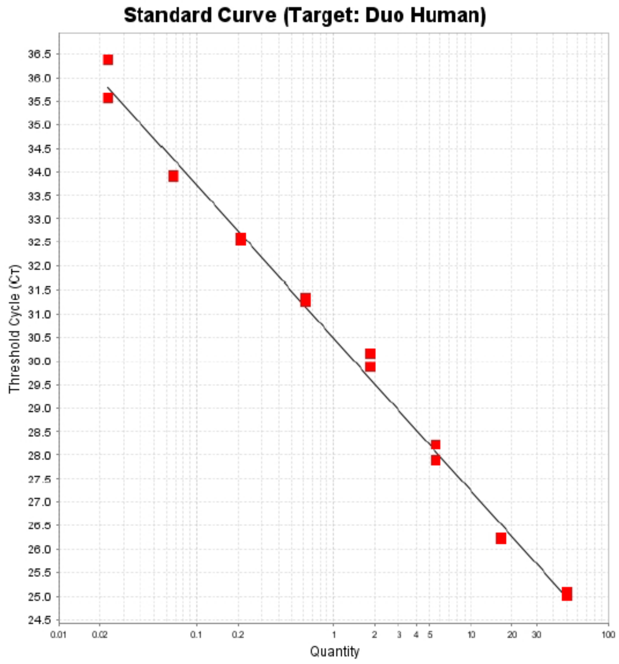

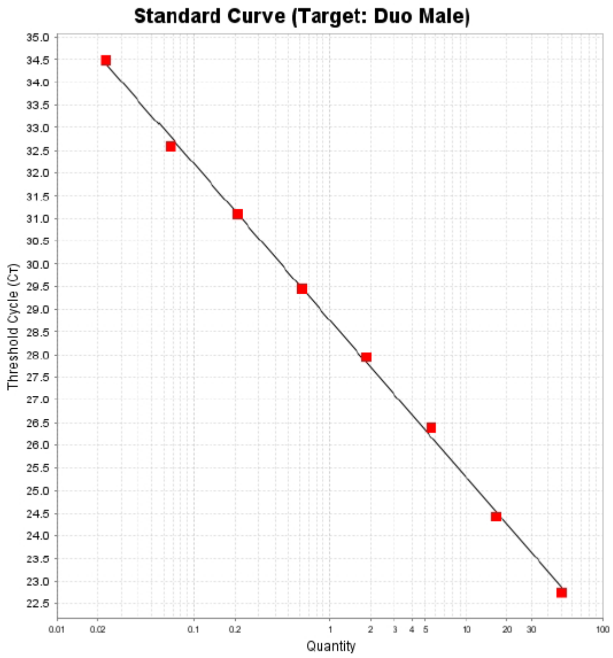

Human standard curve: R^2=0.997, slope = -3.438.

Male standard curve: R^2=0.999, slope = - 3.457.
