# Supplementary material for: Heterogeneous Distribution of Fetal Microchimerism in Local Breast Cancer Environment
Source: PLoS One. 2016 Jan 25;11(1):e0147675. doi: 10.1371/journal.pone.0147675 (PMC4726590; doi:10.1371/journal.pone.0147675)

S2 Figure. Amplification plot for human and male DNA, for standards and samples (example).

Human and male amplification curves. The scale at left shows fluorescence units as generated by the by the 7500 SDS v1.2.3 software of Applied Biosystems. Abscissa, cycle numbers. The readout threshold is indicated by the horizontal red / blue line. No template controls and negative controls generate no signal.

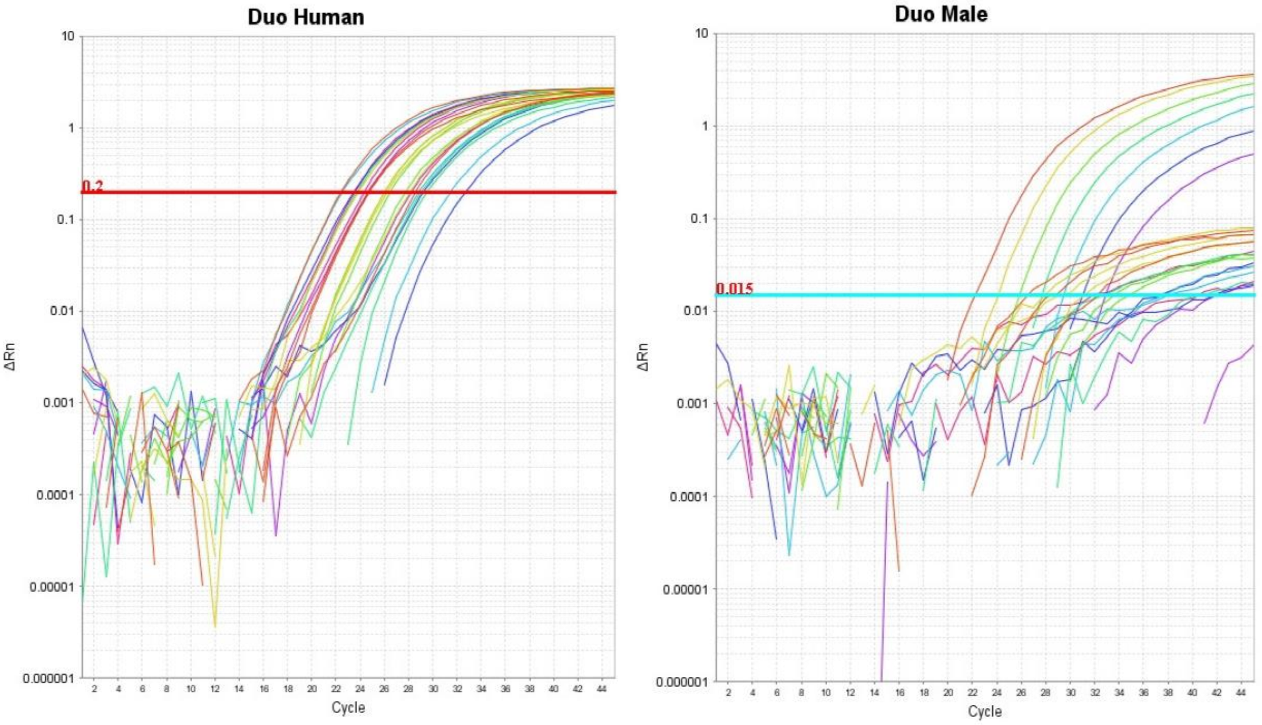

Supplement: S2 Fig — (PDF) [file pone.0147675.s003.pdf]
